# Molecular Binding Mechanism of TtgR Repressor to Antibiotics and Antimicrobials

**DOI:** 10.1371/journal.pone.0138469

**Published:** 2015-09-30

**Authors:** Ana Maria Fernandez-Escamilla, Gregorio Fernandez-Ballester, Bertrand Morel, Salvador Casares-Atienza, Juan Luis Ramos

**Affiliations:** 1 Environmental Protection Department, Estación Experimental del Zaidín (EEZ), Spanish National Research Council (CSIC), C/ Profesor Albareda, 1, E-18008 Granada, Spain; 2 Institute of Molecular and Cellular Biology (IBMC), Miguel Hernández University, Avda/ de la Universidad s/n, E-03202 Elche, Alicante, Spain; 3 Physical-Chemistry Department, Faculty of Sciences, University of Granada, Campus Fuentenueva, E-18071 Granada, Spain; University of Connecticut, UNITED STATES

## Abstract

A disturbing phenomenon in contemporary medicine is the prevalence of multidrug-resistant pathogenic bacteria. Efflux pumps contribute strongly to this antimicrobial drug resistance, which leads to the subsequent failure of clinical treatments. The TtgR protein of *Pseudomonas putida* is a HTH-type transcriptional repressor that controls expression of the TtgABC efflux pump, which is the main contributor to resistance against several antimicrobials and toxic compounds in this microbe. One of the main strategies to modulate the bacterial resistance is the rational modification of the ligand binding target site. We report the design and characterization of four mutants-TtgR^S77A^, TtgR^E78A^, TtgR^N110A^ and TtgR^H114A^ - at the active ligand binding site. The biophysical characterization of the mutants, in the presence and in the absence of different antimicrobials, revealed that TtgR^N110A^ is the variant with highest thermal stability, under any of the experimental conditions tested. EMSA experiments also showed a different dissociation pattern from the operator for TtgR^N110A^, in the presence of several antimicrobials, making it a key residue in the TtgR protein repression mechanism of the TtgABC efflux pump. We found that TtgR^E78A^ stability is the most affected upon effector binding. We also probe that one mutation at the C-terminal half of helix-α4, TtgR^S77A^, provokes a severe protein structure distortion, demonstrating the important role of this residue in the overall protein structure and on the ligand binding site. The data provide new information and deepen the understanding of the TtgR-effector binding mechanism and consequently the TtgABC efflux pump regulation mechanism in *Pseudomonas putida*.

## Introduction

The study of bacterial resistance to antibiotics and therapeutic agents has become a very hot topic due to its implications in human health [1, 2]. The major bacterial cell protective mechanism is the expression of membrane transporters that recognize and actively export toxic substances out of the bacterial cells [3–7]. Multidrug efflux transporters are an essential module in both intrinsic and acquired bacterial resistance to a large number of antimicrobials and organic compounds.

Recent findings have shown the significance of multi-drug efflux pumps in cancer chemotherapy and the treatment of bacterial infections; many efflux pumps can recognize a number of structurally unrelated toxic compounds and actively extrude them from cells [8]. A deeper understanding of the mechanisms that control these pumps is an initial step toward influencing these processes for therapeutic purposes; this could be particularly important in the design of inhibitors, and the development of new therapies.

Some *Pseudomonas putida* species, as well as other bacteria, possess the ability to survive in extremely toxic conditions utilizing several strategies to overcome toxic compounds. *Pseudomonas putida* DOT-T1E shows a high resistance phenotype to diverse xenobiotic and organic compounds such as toluene, flavonoids, β-lactam antibiotics and other antimicrobial agents [9]. The mechanism responsible for its resistance phenotype is the active extrusion of toxic compounds through cell membrane-bound efflux pumps; the expression of the pumps is highly regulated at the transcriptional level [10, 11]. *Pseudomonas putida* DOT-T1E has three homologous efflux pumps (TtgABC, TtgDEF and TtgGHI) with 70% identity at the protein level but significant differences in ligand binding specificities. All of these efflux pumps belong to the RND family of transporters, the most relevant family in respect to resistance to therapeutic agents [5]. The primary contributor to resistance is the TtgABC efflux pump, which is tightly regulated by the transcriptional regulator TtgR (a member of the TetR transcriptional repressor family), which inhibits the transcription of both *ttgABC* operon and control its own expression. The TtgR repressor is a homodimer that binds to its operator located between *ttgR* and the divergent *ttgA* promoters [10]. From a structural point of view, each TtgR monomer consists of two domains, namely the DNA binding domain (from helix-α1 to-α3) and the ligand binding domain (from helix-α4 to-α9) [11]. The ligand binding domain encloses a wide funnel-shaped cavity that confers the multidrug binding potential to the TtgR protein. Binding to the effector induces the dissociation of the repressor-operator complex through helix-α4 which acts as a piston pushing the DNA away from the repressor [12, 13]. The ligand binding domain contains two distinct and overlapping ligand binding sites: the low affinity site mainly made up of hydrophobic residues, situated on the top of the pocket away from the DNA binding site, and a high affinity binding site rich in polar residues, located at the bottom of the cavity [12]. Previous studies on the TtgR multidrug binding potential have shown that it binds with moderate to high affinity to plant-derived compounds such as phloretin and naringenin and with low affinity to chloramphenicol [11]. All of these compounds are characterized by having an aromatic ring [13]. A recent study has described a new microbial biosensor, based on the TtgR's ability to bind unrelated structural compounds, to detect toxic compounds [14]. This current study investigates the biophysical characterization of four mutations in the TtgR active binding site (TtgR^S77A^, TtgR^E78A^, TtgR^N110A^ and TtgR^H114A^) and aims to further define the key role of these residues. This information will be highly beneficial and provide insight into the molecular mechanisms that govern the expression of the TtgABC efflux pump.

## Materials and Methods

### Site-directed mutagenesis, overexpression and purification of His-tagged TtgR^WT^ and mutants

The native *ttgR* structural gene was cloned into pET29a(+) expression vector. TtgR mutants were generated by amplification of the above construction using the MBLong PCR Kit (Dominion MBL) and the primers which incorporated the mismatch(es) to introduce the desired mutation. The PCR product was transformed in *E*. *coli* BL21(DE3) strain. The sequence of each mutant was confirmed by DNA sequencing. *E*. *coli* BL21(DE3) cells were grown in 300 ml of 32Y rich culture medium in 2L conical flasks with 50 μg/ml kanamycin, at 37°C, shaking at 200 rpm until an optical density at 660 nm of 0.5–0.6 was reached. At this time, the temperature was decreased to 18°C and protein expression was induced with 1mM of isopropyl β-D-thiogalactopyranoside (IPTG). The culture was incubated overnight. Cells were harvested by centrifugation (15 min, 4,400 x g, 4°C) and the pellet was resuspended in 45 mL of buffer 20 mM sodium phosphate pH 7.4, 500 mM NaCl, 10 mM imidazole, 5% (v/v) glycerol, 0.1 mM EDTA supplemented with 1μl Benzonase (100000U) enzyme and half a tablet of COMPLETE R protease inhibitor (ROCHE) per 10 mL of resuspended solution. The cells were sonicated and the extract was centrifuged 38,000 x g for 1 hour at 4°C. The supernatant was filtered and loaded onto a 5 ml HisTrap HP column (GE Healthcare) and eluted using an imidazole gradient (10 to 500 mM). Eluted fractions of TtgR were exhaustively dialyzed at 4°C overnight against buffer: 25 mM pipes pH 7.0, 250 mM NaCl, 10 mM KCl, 10 mM magnesium acetate, 0.1 mM ethylenediaminetetraacetic acid (EDTA), 1 mM tris(2-carboxyethyl)phosphine (TCEP), 5% (v/v) glycerol and storage at 70°C. Protein concentrations were determined spectrophotometrically by UV absorption at 280 nm using an extinction coefficient of 20970 M^-1^ cm^-1^, determined via ProtParam algorithm (http://web.expasy.org/protparam/) based on the amino acid sequence.

### Circular dichroism (CD)

CD experiments were performed using a Jasco J-715 (Tokyo, Japan) spectropolarimeter equipped with a thermostatically controlled cell holder. Measurements of far-UV CD spectra were recorded at different temperatures with a 0.1 cm path-length quartz cuvette using 1 nm bandwidth, 100 nm.min^-1^ scan rate, 1 second response time and 5 scans average. In thermal melting experiments, the CD signal was monitored as a function of temperature at 222 nm (the negative band characteristic of α-helix spectrum). As described previously by Greenfield and co-workers [15], melting temperatures were determined by calculating the single derivative of the curves. The maxima were at the midpoints of the folding transitions. Data analysis was performed using Microcal Origin software (Originlab, Northampton, MA, USA). Baselines obtained from samples containing only buffer were subtracted from all the data reported.

### Differential scanning calorimetry (DSC)

DSC experiments were carried out using a VP-DSC (Valerian-Plotnikov differential scanning calorimeter), capillary-cell microcalorimeter from MicroCal (Northampton, MA) at 90°C/h scan rate from 5°C to 85°C. Calorimetric cells (operating volume 0.133 mL) were kept under an excess pressure of 60 psi bar to prevent degassing during the scan. Several buffer-buffer baselines were obtained before each run with protein solution in order to ascertain proper equilibration of the instrument. Protein samples were exhaustively dialyzed at 4°C overnight against buffer: 25 mM pipes pH 7.0, 250 mM NaCl, 10 mM KCl, 10 mM magnesium acetate, 0.1 mM ethylenediaminetetraacetic acid (EDTA), 1 mM *tris*(2-carboxyethyl)phosphine (TCEP), 5% (v/v) glycerol. The protein concentrations used were: TtgR^WT^ (c = 38.2 μM), TtgR^E78A^ (c = 44.7 μM), TtgR^N110A^ (c = 38 μM), TtgR^H114A^ (c = 41.7 μM), TtgR^S77A^ (c = 14.4 μM). A concentration of 250μM of the effector was added in the interaction experiments.

### Electrophoretic mobility shift assays (EMSA)

A 189-bp DNA fragment containing the *ttgABC-ttgR* intergenic region was obtained by PCR from the *P*. *putida* DOT-T1E chromosome as described previously [10]. This DNA probe was radiolabeled (1 nM, ≅10,000 cpm) and incubated with the appropriate concentrations of purified variants of TtgR (2 μM). The protein was dissolved in 10 μl of DNA binding buffer (10 mM Tris-HCl, pH 7.0, 250 mM NaCl, 10 mM magnesium acetate, 10 mM KCl, 5% glycerol (v/v), 0.1 mM EDTA and 5 mM dithiothreitol (DTT) supplemented with 20 μg/ml of polyd(I-C) and 200 μg/ml of Bovine Serum Albumin (BSA)). The stock solutions of different effectors were prepared in 100% dimethyl sulphoxide (DMSO) and diluted into the binding reaction at a final concentration of 5μM. Reactions were incubated for 10 min at 30°C and electrophoresed on 4.5% (w/v) native polyacrylamide gels (Bio-Rad Mini-Protean II) at 50 volts for 2h in Tris glycine buffer (25 mM tris-HCl, pH 8.0, 200 mM glycine). The gels were dried and the results were analyzed with a GS525 molecular imager (Bio- Rad) and QuantityOne 1-D analysis version 4.6.2 software (the Discovery series, Bio-Rad).

### Molecular modeling of variants

Crystal structures of TtgR, in the presence of effectors, were downloaded directly from RCSB Protein Data Bank (http://www.rcsb.org). The TtgR-effector complexes used were: chloramphenicol (PDB ID 2UXP at 2.7 Å resolution), naringenin (PDB ID 2UXU at 2.3 Å), and phloretin (PDB ID 2UXI at 2.5 Å). Since, the crystal structure of TtgR, in the absence of effectors, was not available, we instead used the mutant TtgR H67A (PDB code 2XDN) at 2.2 Å resolution. The mutation was reverted to His and used as wild type conformation (WT). Mutagenesis and stability energy measurements were performed using FoldX [16, 17] on the CRG site: http://foldx.crg.es. The force field of FoldX allowed us to evaluate the properties of the structure. Parameters such as atomic contact map, accessibility of the atoms and residues, backbone dihedral angles, hydrogen bonds and electrostatic networks of the protein were assessed. Binding energy measurements were performed with Amber03 [18] implemented in Yasara [19, 20] (http://www.yasara.org).

### Isothermal Titration Calorimetry (ITC)

ITC experiments were performed with a high-sensitivity VP-ITC (Valerian-Plotnikov isothermal titration calorimeter, MicroCal, Northampton, MA) at 30°C. Protein samples were extensively dialyzed against buffer 25 mM Pipes, 250 mM sodium chloride, 10 mM magnesium acetate, 10 mM potassium chloride, 0.1 mM EDTA, 1 mM DTT and 5% (v/v) glycerol, pH 7.0 for 24 hours at 4°C prior to the experiments. Due to the intrinsic low solubility of effectors in water, stock solutions were prepared by dissolving the available commercial powder of phloretin, naringenin and chloramphenicol (Sigma-Aldrich) directly in 100% Dimethyl sulphoxide (DMSO) up to a final concentration of 500 mM. Effector samples were prepared by direct dilution of the stock solutions in the protein dialysis buffer, always keeping DMSO concentration under 0.2% (v/v) in the final effector solution sample. DMSO was also added to the protein solutions up to the same final concentration, 0.2% (v/v). Possible pH variations were monitored and corrected prior to the experiment. Protein and effector concentrations were determined spectrophotometrically by UV absorption: TtgR variants at 280 nm using an extinction coefficient of 20970 M^-1^ cm^-1^, determined via ProtParam algorithm, chloramphenicol at 278 nm using an extinction coefficient of 9628 M^-1^ cm^-1^ [21] and naringenin at 290 nm using an extinction coefficient of 17600 M^-1^ cm^-1^ [22]. The stock solution of phloretin at 500 mM was prepared in DMSO and subsequently diluted with dialysis buffer to the desired final concentration. Both, protein and effector solutions were degassed for 10 min before measurements. The TtgR protein solutions in the calorimetric cell (12–15 μM) were titrated with the corresponding effector solution (0.3–3 mM) in a series of identical successive injections of 5 μL each. The heats derived from the dilution of the effectors into the buffer were determined in independent blank experiments and subtracted from raw titration data before proceeding with the data analysis using Origin data analysis software (Originlab, Northampton, MA, USA).

## Results

### Rational design of TtgR binding site mutants

**Fig 1:**
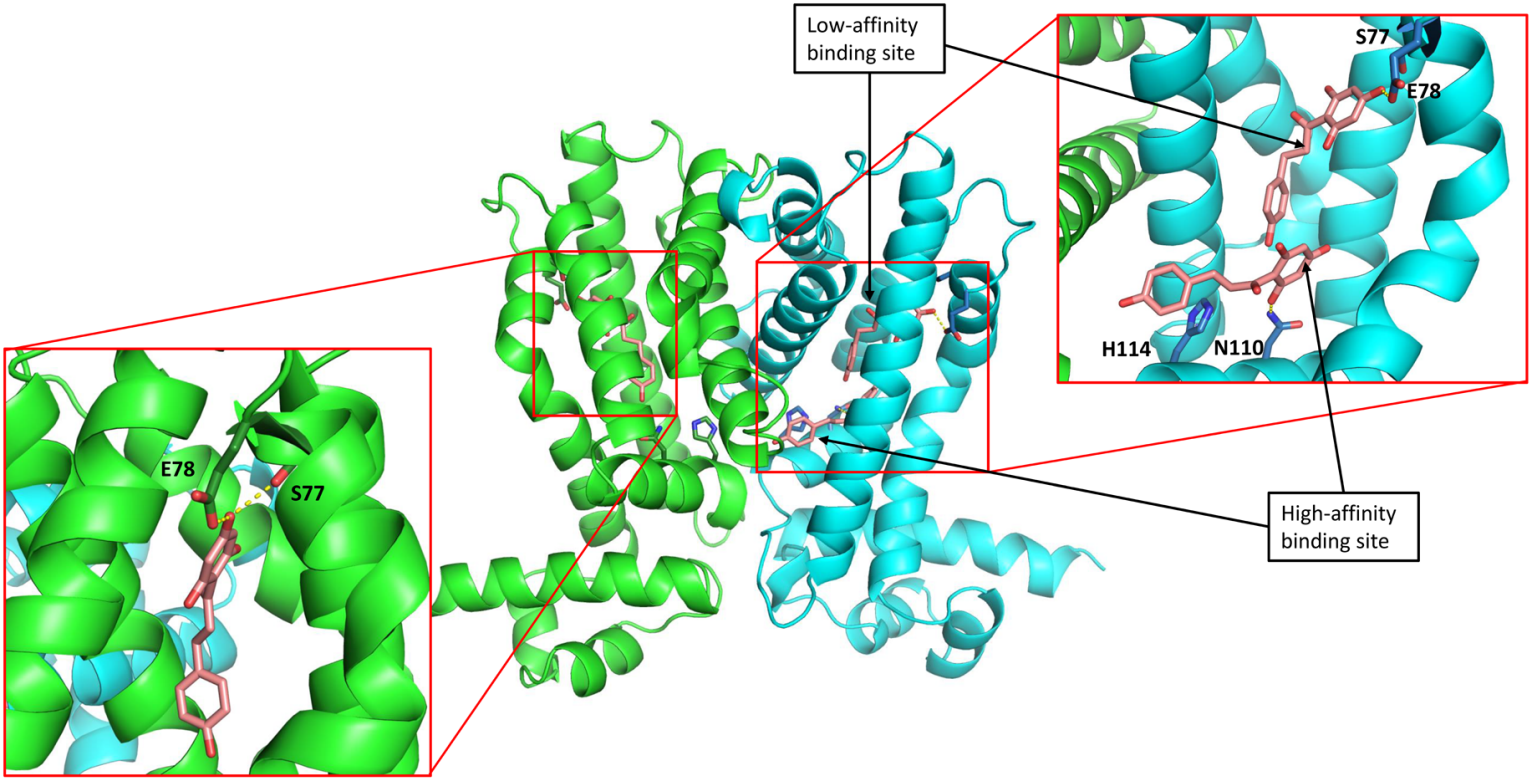
Central panel shows a global view of TtgRWT in complex with phloretin (PDB code 2UXI). Top panel shows a zoom on the binding sites of one monomer containing two molecules of phloretin, bound to low- and high-affinity binding sites. Residues 122–154 in alpha-helix conformation have been hidden for clearly show the biding site. Bottom panel shows a third phloretin molecule bound to the low affinity binding site of the second monomer. Discontinuous, yellow lines denote phloretin hydrogen bond formation. In all panels the mutated residues are highlighted.

The TtgABC efflux pump expression takes place in response to the binding of toxic compounds directly to the TtgR dimer in *Pseudomonas putida* DOT-T1E. These compounds differ in structure one from another. The importance of some TtgR residues in the ligand-protein interaction have previously been reported (e.g. Arg176, whose mutation to Gly reduced the affinity for phloretin) [12]. It has also been shown that several other mutations altered the affinity of TtgR for its operator [13]. These previous studies provide insufficient data to propose a detailed description of the interactions in the active binding pocket upon ligand binding. To determine critical structural interactions in the TtgR binding process, we performed a deeper examination of the crystallographic structures of the TtgR protein in complex with several natural ligands. We then rationally designed TtgR variants mutated in essential residues involved in the binding process and selected three ligands for the study of regulator/effector interactions, including two plant secondary metabolites (naringenin and phloretin), and an antibiotic (chloramphenicol). These ligands have different methods of interaction with the residues located at the most hydrophobic region of the TtgR binding pocket. A single molecule of chloramphenicol and naringenin are bound per protein monomer, these compounds bind at the high affinity site in a very similar position, with the protein-ligand interactions specific for each effector. Remarkably, phloretin is the only organic compound capable to bind, at the same time, to the high and low affinity binding sites [12] (Fig 1). The crystallographic structural analysis of the TtgR complex with phloretin and naringenin stressed the importance of the polar residues Asn110 and His114 located in the lower part of the binding pocket at helix-α6. This part of the vast cavity corresponds to the high affinity area, near to the repressor binding site and is mostly formed by polar residues (Asn110, His114 and Asp172). Upon binding of phloretin or naringenin, Asn110 is positioned very favorably to be coordinated to the amino and dimethylamino effector groups (Fig 1 top panel). In fact, Asn110 has been proposed, using docking analysis, as a ligand-sensor residue [23]. His114 is one of the polar residues at the high affinity site, away from the ligand compared to Asn110, which plays a putative ‘assistant’ role in the correct positioning of the ligand. In addition to the hydrophobic interactions described for the low affinity binding pocket [12], two residues belonging to helix-α4, Ser77 and Glu78, appeared to play a major role, not only in regard to the ligand binding but also to maintain the global structure of the protein (Fig 1 bottom panel). To get more information about the binding site constraints, it is essential to perform a comparative study of WT crystal structure and its complexed forms. TtgR^WT^ crystal structure has not been solved, this is due to the poor diffraction of the crystals obtained in absence of ligands; this finding indicates a more flexible structure. For this reason, we modeled the TtgR^WT^ structure using the crystal coordinates of TtgR^H67A^ whose mutation was reverted to His (see Materials and Methods section). This approximation is based on the absence of appreciable conformational changes between TtgR^H67A^ and TtgR^WT^ upon ligand binding effectors of different nature. Using the structural information described above we aimed to explore the importance of these residues on the binding mechanism; for this, we selected and mutated Ser77, Glu78, Asn110 and His114 residues to Ala.

### Structural and thermal effects of binding site mutations on TtgR

**Fig 2:**
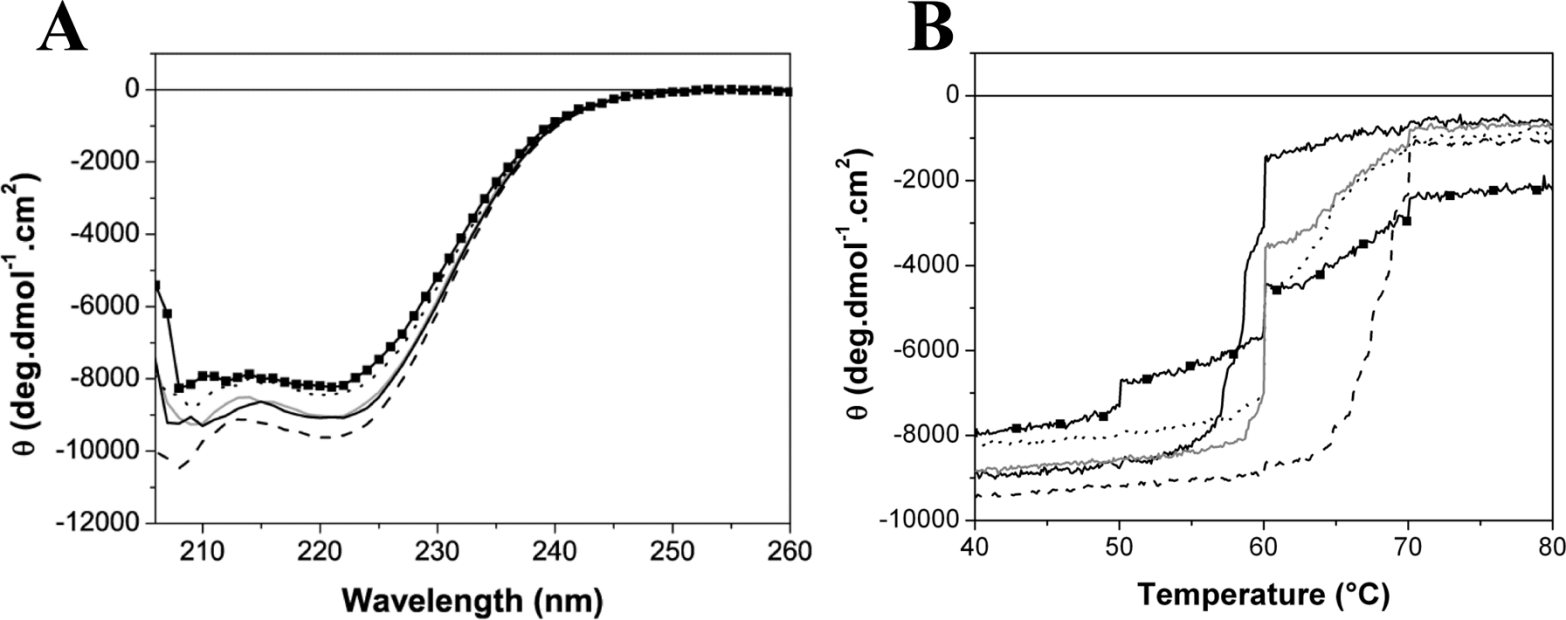
Far UV CD experiments of TtgR^WT^ and variants in the absence of effectors. (A) Far UV CD spectra at 30°C. (B) Thermal unfolding of TtgR^WT^ and mutants monitored by CD ellipticity at 222 nm. Variants are represented in grey line (TtgR^WT^), black solid line (TtgR^H114A^), black dashed line (TtgR^N110A^), black dotted line (TtgR^E78A^) and black squares and line (TtgR^S77A^).

To analyze the conservation of the secondary structure of the variants, we carried out far-UV CD experiments on the native protein and mutants at 30°C (Fig 2A). The resulting spectra are similar, indicating that the overall secondary structure is not altered by mutations. The CD spectra were deconvoluted using the CDNN program, http://bioinformatik.biochemtech.uni-halle.de/cdnn/ [24], in order to determine the protein percentage in α-helix, beta-turn and random coil. The data revealed a similar percentage of α-helix, beta-turn and random coil for most of the variants, in comparison with TtgR^WT^ (Table 1).

**Table 1:** Structural contents of TtgR ^WT^ and mutants (%), in the absence of effectors. Data were obtained by deconvolution of the far UV CD spectra at 30°C.

	WT	H114A	E78A	S77A	N110A
	210–260 nm				
Helix	59.5	61	60.6	53.3	59.8
Antiparallel	3.7	3.5	3.5	4.5	3.6
Parallel	4.5	4.3	4.4	5.2	4.4
Beta-Turn	12.9	12.7	12.7	13.7	12.8
Rndm. Coil	19.3	18.6	18.8	22.1	19.1

The effect of mutations on protein stability was examined by means of residue molar ellipticity changes at 222 nm as a temperature function (Fig 2B). For most of the variants, the results showed sigmoidal plots suggesting a single denaturation event. After heating up to 90°C, the proteins precipitated into white particles. This fact demonstrates the non-reversibility of the unfolding process. It is common for most mesophilic proteins to suffer some degradation of their chemical structure decreasing the reversibility of the process by heating above 70°C [25]. Nevertheless, the TtgR^S77A^ unfolding plot showed more than one transition, pointing to a possible aggregation process induced by temperature since the molar ellipticity value remained unchanged until it was at more than 50°C. The CD denaturation temperatures (Tm^CD^) for WT and variants ranged from 58.6 to 68.3°C (Table 2, effector free). Notably, Tm^CD^ is higher for E78A and N110A mutations when compared to WT indicative of protein stabilization while H114A showed protein destabilization with lower Tm^CD^.

**Table 2:** Thermodynamic parameters obtained by DSC and CD. Thermodynamic parameters for the thermal unfolding of TtgR^WT^ and variants (TtgR^H114A^, TtgR^N110A^, TtgR^E78A^ and TtgR^S77A^) obtained from DSC and far-UV CD measurements. Internal calibrations of the CD and DSC equipments give errors in Tm of ± 0.20°C and ± 0.10°C respectively.

	WT			N110A			H114A			E78A		
	Tm^CD^	Tm^DSC^	ΔHm	Tm^CD^	Tm^DSC^	ΔHm	Tm^CD^	Tm^DSC^	ΔHm	Tm^CD^	Tm^DSC^	ΔHm
	(°C)	(°C)	(kJ.mol^-1^)	(°C)	(°C)	(kJ.mol^-1^)	(°C)	(°C)	(kJ.mol^-1^)	(°C)	(°C)	(kJ.mol^-1^)
Effector free	60.10	60.69	183±7	68.30	68.53	335±12	58.60	57.13	226±15	62.10	62.63	98±13
Chloramphenicol	64.10	63.92	204±21	68.30	68.38	364±18	60.20	60.36	254±7	64.95	65.22	149±7
Naringenin	68.60	67.83	296±13	69.80	69.78	445±16	64.90	63.77	305±3	69.90	69.86	191±13
Phloretin	69.80	69.55	288±10	71.80	71.89	437±2	66.90	65.78	300±23	69.90	68.65	175±8

**Fig 3:**
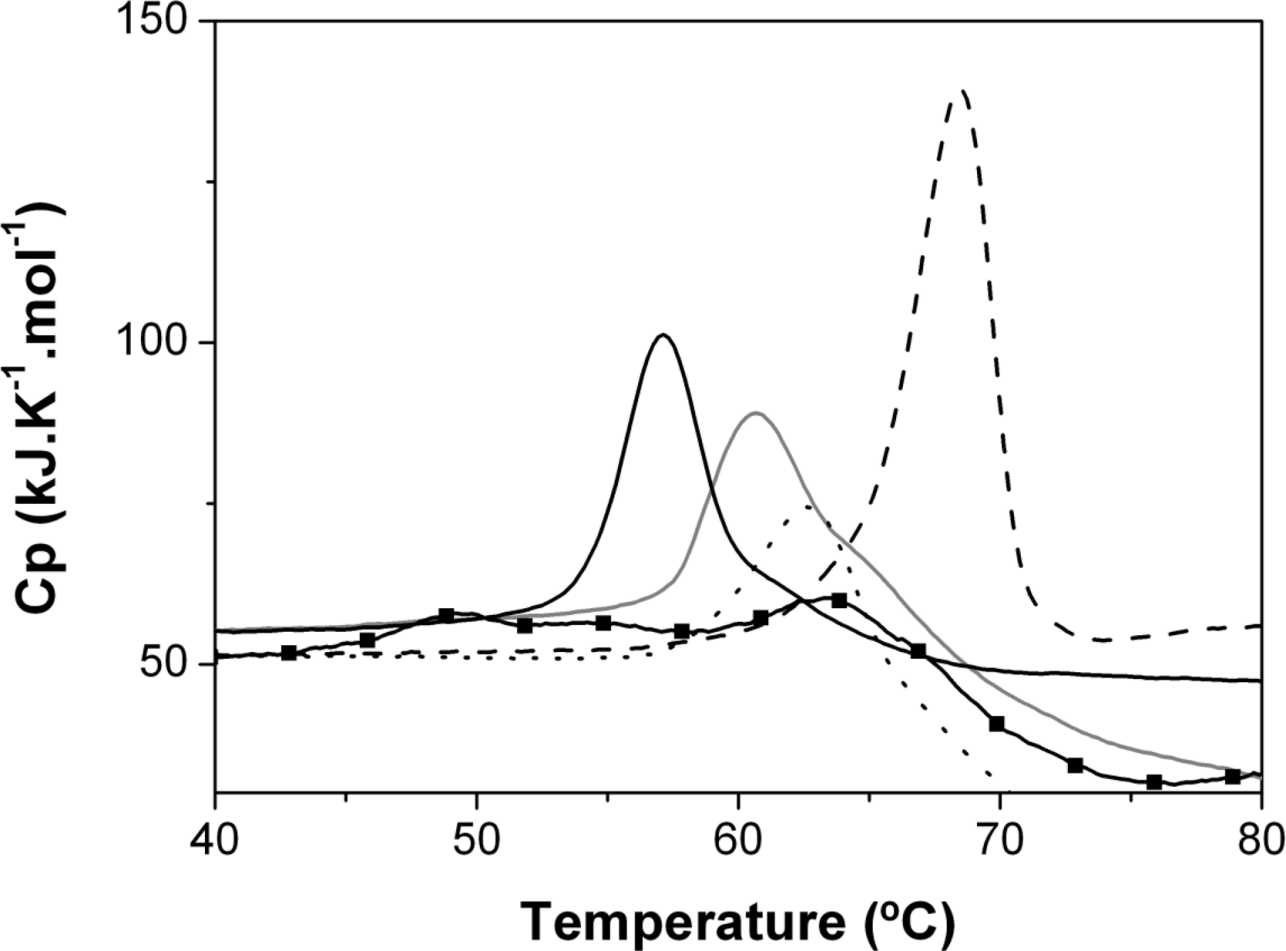
Thermal denaturation of TtgR^WT^ and its variants in the absence of effectors, followed by Differential Scanning Calorimetry (DSC). TtgR^WT^ (grey line), TtgR^H114A^ (black solid line), TtgR^N110A^ (black dashed line), TtgR^E78A^ (black dotted line) and TtgR^S77A^ (black squares and line).

However, the complex nature of the unfolding process and the thermally induced precipitation of the samples make the Tm determination for some of the mutants quite difficult using a spectrophotometric technique. For these reasons, thermodynamic protein stability changes produced by mutations were also investigated using Differential Scanning Calorimetry (DSC) (Fig 3).

The thermal denaturation experiments showed that TtgR^WT^ and most of the variants unfold in a single event at melting temperatures (Tm^DSC^) between 57.13°C to 68.53°C (Table 2, effector free). Analysis of the DSC scans revealed a destabilizing effect produced by mutation of H114A and stabilizing effects for the N110A and E78A mutations, this was much more pronounced in the case of N110A (Fig 3 and Table 2). These values are almost equal to those obtained by thermal denaturation followed by circular dichroism signal at 222 nm (Fig 2B and Table 2). It is worth mentioning that mutation S77A showed more than one unfolding transition with a broad peak and low unfolding enthalpies, revealing protein aggregation induced by temperature that is in full accordance with the CD data described above.

Additionally, we measured the theoretical stability energies of the modelled TtgR^WT^ and mutants (TtgR^S77A^, TtgR^E78A^, TtgR^N110A^ and TtgR^H114A^) by means of FoldX [17]. The analysis of the energy values (ΔΔG in kcal/mol) denoted an increase in stability energy for E78A and N110A mutants, and pronounced stability decreases in H114A and S77A (see Table 3, effector free) again, in good agreement with the CD and DSC experiments (see next section for more details)

**Table 3:** Theoretical energy measurements of models.

ΔΔG (kcal/mol)*	Effector free	Phloretin	Naringenin	Chloramphenicol
WT	0	0	0	0
E78A	-1.59	1.19	-0.93	0.52
H114A	2.65	0.37	0.37	0.30
N110A	-0.96	-0.01	-2.18	0.31

*The values are presented in ΔΔG (kcal/mol) as the difference between the free energy of mutants with respect to the free energy of wild type. **Effector free**: stability energy calculated as the difference between folded and unfolded states. **With effectors**: free energy of binding calculated as the difference between the bound and unbound state.

### Effects of the mutations on the TtgR binding process

**Fig 4:**
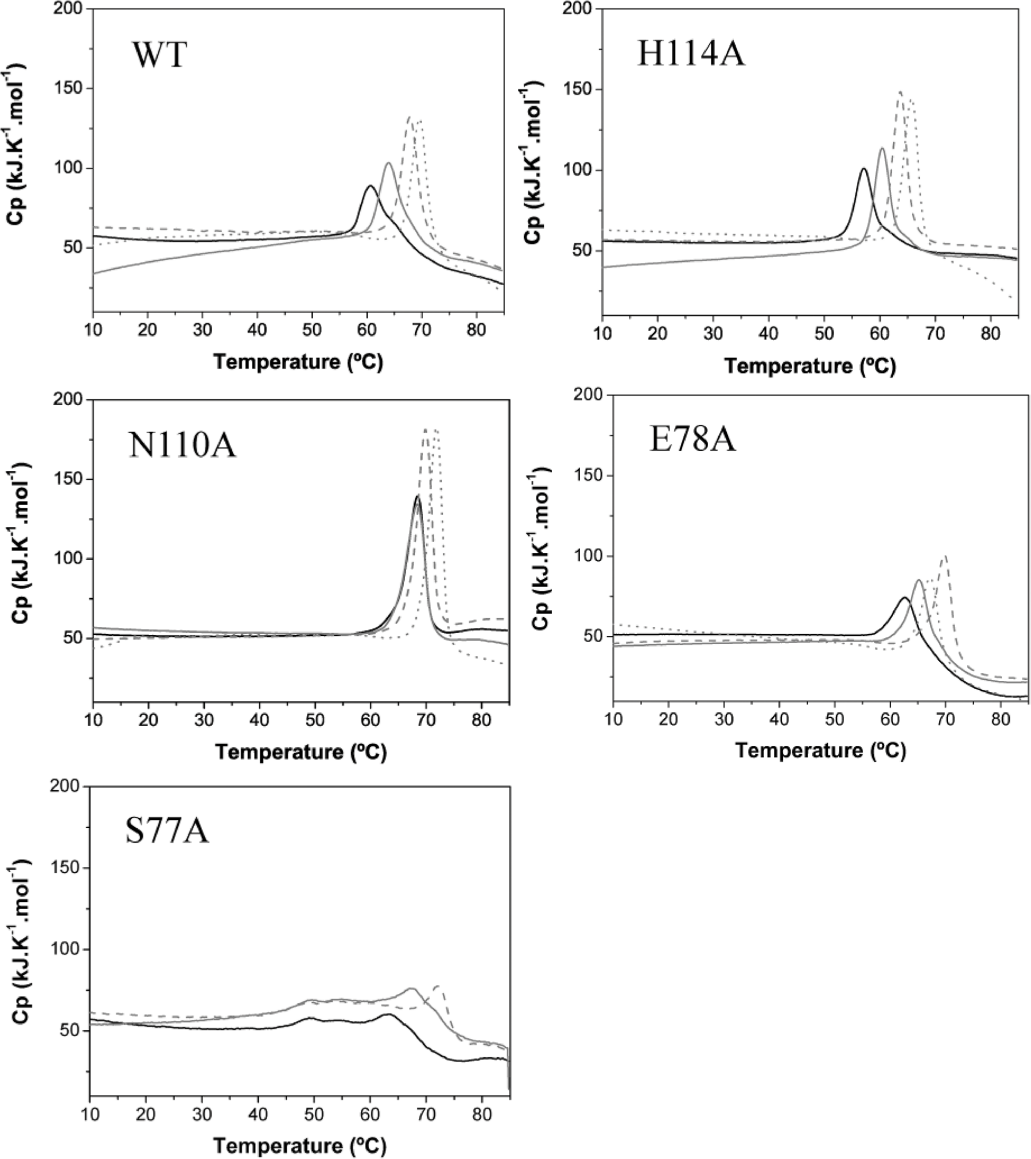
Thermal denaturation followed by DSC of TtgR^WT^ and variants (TtgR^H114A^, TtgR^N110A^, TtgR^E78A^ and TtgR^S77A^) in the presence and in the absence of ligands (black solid lines free; grey solid lines 250 μM of chloramphenicol; grey dashed lines 250 μM naringenin; grey dotted lines 250 μM phloretin).

To characterize the binding of the effectors to TtgR^WT^ and its variants as well as to describe the extent of the effect of each ligand on the structure and stability of the proteins, we performed temperature induced unfolding experiments on all of the variants using DSC and CD in the presence of naringenin, phloretin and chloramphenicol. The secondary structure of the protein variants bound to effectors was measured by Far-UV CD at a ligand concentration of 250 μM. The spectra indicated that the TtgR^WT^ and variant conformations are not altered by the presence of effectors (data not shown). In general, the CD signal monitoring at 222 nm as a function of temperature, showed a significant thermal stabilization upon ligand binding (Table 2). Equivalent thermal unfolding experiments using DSC also revealed that TtgR^WT^ and its mutants are more stable when complexed with the effectors (Table 2, Fig 4). In DSC experiments, the rise of Tm (effector free < Chlr < Nar < Phlr) was concomitant with the increase of the unfolding enthalpic contribution to the protein stability (Table 2) as shown by the enlarged area under the peak, which can be interpreted as an indication of the protein stabilization. Phloretin is the most stabilizing effector, closely followed by naringenin, while the less stabilizing effector is chloramphenicol. The highest Tm was measured for the N110A variant and its thermal stability was less affected by the binding. In the presence of any effector, the less stable variant is H114A. Particularly, the stability of E78A is most dependent on effector binding, being more stable when bound to naringenin and less stable bound to phloretin.

We also measured the theoretical free energy of binding for all variants in the presence of effectors, by means of Amber03 [26]. The variation of free energy was calculated as the difference between the energy of mutants compared to the wild type structural model, in the presence or absence of effectors. In the absence of effectors, we calculated the energy as the difference between folded and unfolded states. In the presence of effectors, the free energy of binding was calculated as the difference between bound and unbound states. Thus, negative values indicate a strong interaction of the variant and the effector. Table 3 shows the free energy values obtained for variants and effectors. The results clearly identified H114A as the least stable, and N110A as the most stable variants, in good agreement with the experiments described above. The strongest effect of ligand binding was observed for naringenin, closely followed by phloretin. Chloramphenicol behaved as the poorest effector, again in good agreement with the DSC and CD results. Naringenin showed stabilization for E78A and N110A, and almost no effects in other variants, suggesting that these mutations affected ligand binding and/or accessibility of ligand to the pockets. In contrast, phloretin showed negligible stabilization for N110A and destabilization for E78A and H114A. This discrepancy could be due to the method used to measure the theoretical energy, since we neglected cooperative interactions between phloretin molecules and high or low affinity binding sites.

**Fig 5:**
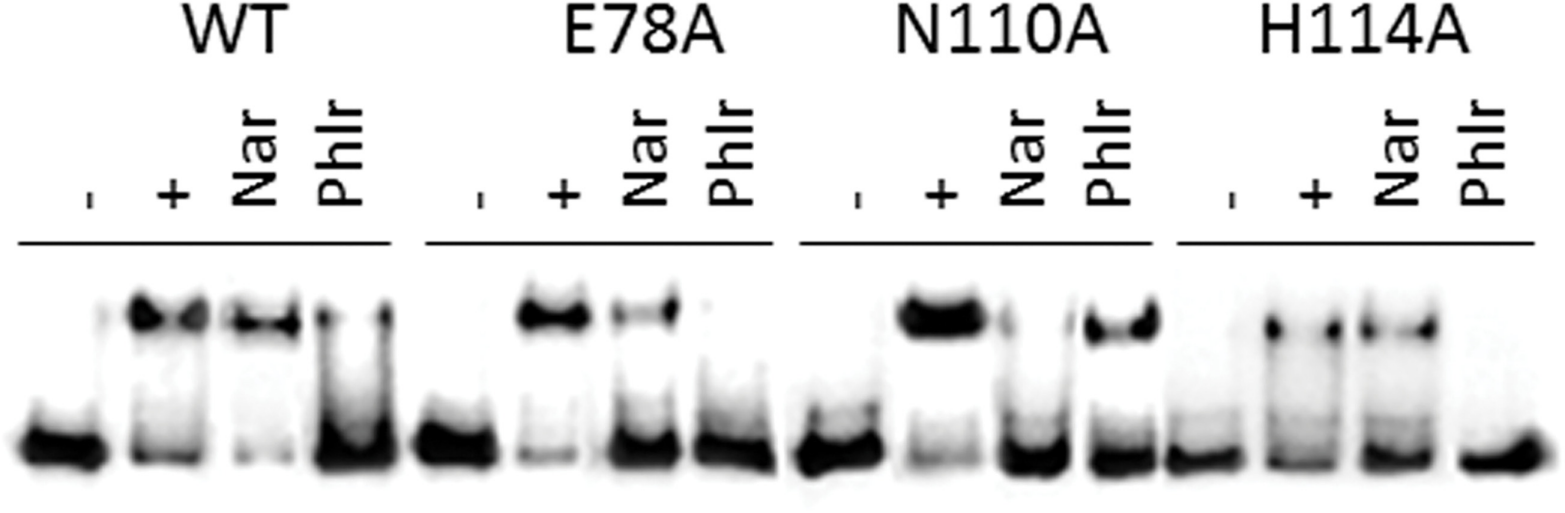
Effect of naringenin and phloretin on the dissociation of TtgR^WT^ and variants (TtgR^H114A^, TtgR^N110A^ and TtgR^E78A^) from *ttgR-ttgABC* intergenic region by EMSA. (-) 1nM of free labeled operator DNA, (+) DNA-complex with 2 μM of TtgR^WT^ and its variants, and DNA complex in the presence of 5μM of naringenin (Nar) and phloretin (Phlr).

As expected, the binding of the effector to the TtgR repressor induced the dissociation of the repressor-operator complex. It has previously been described that phloretin and naringenin cause the dissociation of the repressor-operator complex [13]. To evaluate the effects of the different ligands on the effector-mediated DNA operator dissociation, we performed electrophoretic mobility shift assays (EMSA) (Fig 5). Our results revealed that, in absence of effectors, most of the variants (except TtgR^S77A^) were able to bind to and retard the DNA fragment, containing the *ttgABC-ttgR* intergenic region. In the presence of phloretin, TtgR^E78A^ and TtgR^H114A^ released the DNA completely while TtgR^N110A^ only dissociated 66% of DNA. In the case of naringenin, TtgR^E78A^ and TtgR^H114A^ released around 30% of DNA while TtgR^N110A^ was completely dissociated from the complex. These results suggest that mutations affect the binding or the accessibility of the effectors to the binding pocket and subsequently produce an effect on the repressor-operator complex dissociation.

**Fig 6:**
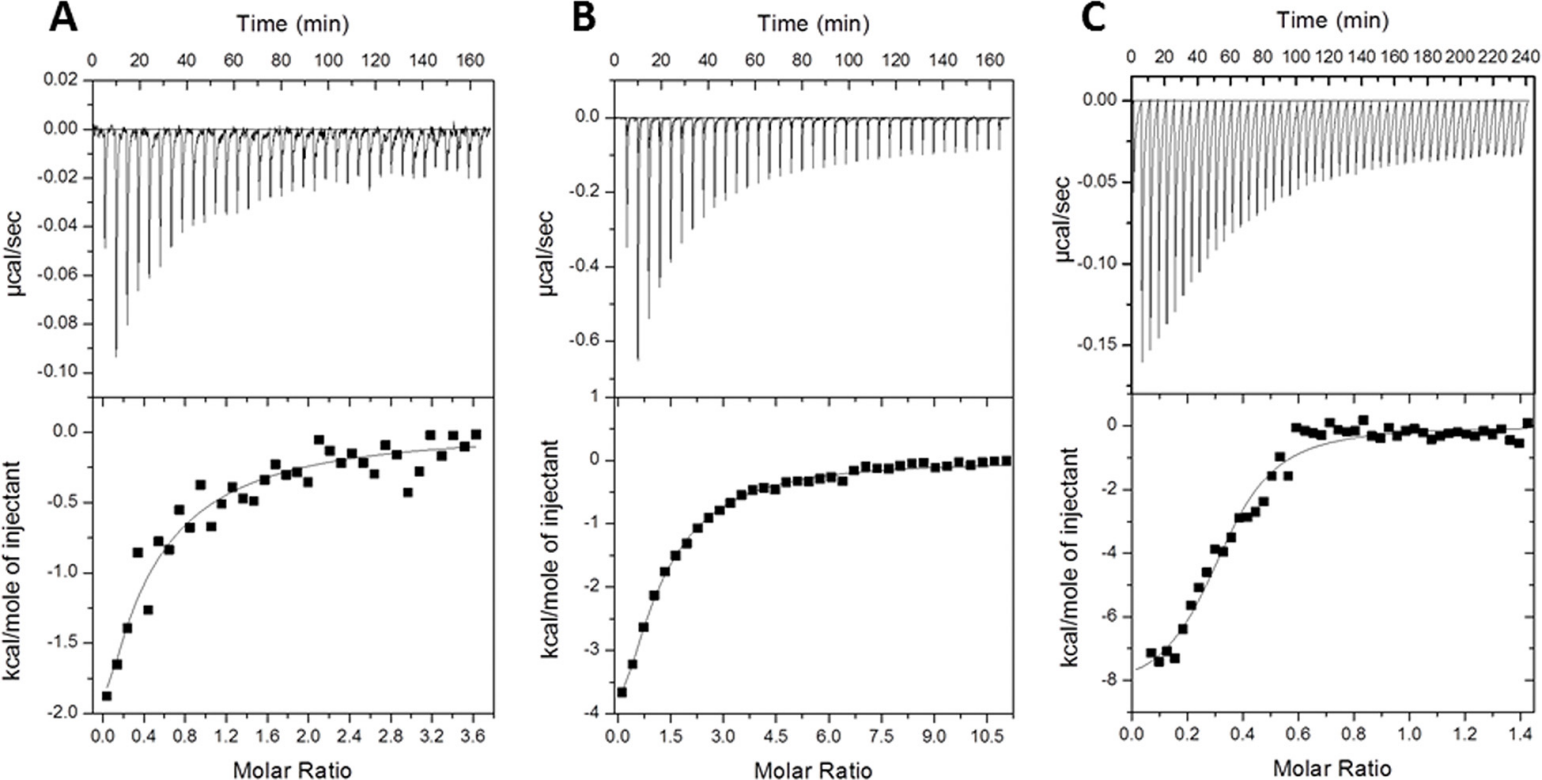
ITC isotherms for the binding of TtgR^E78A^ mutant to: A) chloramphenicol, B) naringenin and C) phloretin. Experiments were performed as described in “Materials and Methods section” at 30°C with injections of the effector diluted in dialysis buffer into the protein. Upper panels: Heats changes for injections. Lower panels: Experimental heats measured for each effector injection. The curves correspond to the best fit using “one type of sites” model.

To quantify the effect of the mutations on the binding process, we performed interaction assays of the variants and effectors using Isothermal Titration Calorimetry (ITC). Thermodynamic parameters were obtained from the analysis of ITC profiles using the “one set of sites” algorithm (Table 4) being consistent with previously published data for TtgR^WT^ [11–13]. The maximum affinity for all of the variants was observed for phloretin followed by naringenin and chloramphenicol. Typical ITC data of TtgR^E78A^ and the three effectors are shown in Fig 6. As mentioned, TtgR^WT^ and its mutants revealed the highest affinity for phloretin with K_D_ values ranging from 0.7 to 1.5 μM (Table 4). In particular, TtgR^E78A^ showed the maximum affinity with a smaller K_D_ value. Most interestingly, the mutation not only affects the binding affinity for the effector but also alter the protein ability to release from its operator.

**Table 4:** Thermodynamic parameters of the association of TtgR and mutants with chloramphenicol, naringenin and phloretin.

Protein	Effector	K_A_	K_D_	ΔG_A_	ΔH_A_
		(M^-1^)	(μM)	(kcal.mol^-1^)	(kcal.mol^-1^)
	Chloramphenicol	(1.8±0.1) x10^5^	5.6±0.4	-7.28±0.04	-5.9±0.2
**WT**	Naringenin	(6.0±1.0) x10^4^	16.0±2.0	-6.66±0.09	-15.0±5.0
	Phloretin	(6.6±0.8) x10^5^	1.5±0.2	-8.07±0.07	-51.1±12.0
	Chloramphenicol	(5.4±0.5) x10^4^	18.5±1.0	-6.57±0.06	-10.8±0.5
**E78A**	Naringenin	(4.6±0.1) x10^4^	21.8±0.6	-6.46±0.02	-9.4±0.1
	Phloretin	(1.4±0.2) x10^6^	0.7±0.1	-8.51±0.11	-8.6±0.4
	Chloramphenicol	(6.4±0.7) x10^4^	15.6±2.0	-6.67±0.07	-6.7±0.4
**N110A**	Naringenin	(5.5±0.3) x10^4^	18.1±1.0	-6.58±0.03	-8.5±0.2
	Phloretin	(9.6±0.7) x10^5^	1.0±0.1	-8.30±0.05	-13.7±0.2
	Chloramphenicol	(1.2±0.7) x10^4^	8.5±0.5	-7.03±0.03	-11.6±0.3
**H114A**	Naringenin	(4.6±0.2) x10^4^	22.0±0.8	-6.46±0.02	-12.2±0.2
	Phloretin	(7.6±0.8) x10^5^	1.3±0.1	-8.15±0.06	-10.8±0.3

Therefore, it would be logical to expect that smaller K_D_ values favour DNA dissociation. EMSA experiments performed to measure the DNA-protein interaction, have confirmed that TtgR^E78A^ was completely released from its operator in the presence of phloretin. On the contrary, most of the variants presented a drastic reduction in their affinity for chloramphenicol. Specifically, K_D_ values for TtgR^E78A^ and TtgR^N110A^ increased more than 3.5 fold in comparison to TtgR^WT^, this result was less pronounced for TtgR^H114A^. These results explain the inability of obtaining EMSA results in the presence of chloramphenicol. In the case of naringenin, the affinities of the variants slightly decrease in regard to TtgR^WT^. Furthermore, TtgR^E78A^ and TtgR^N110A^ presented similar affinities for chloramphenicol and naringenin.

## Discussion

**Fig 7:**
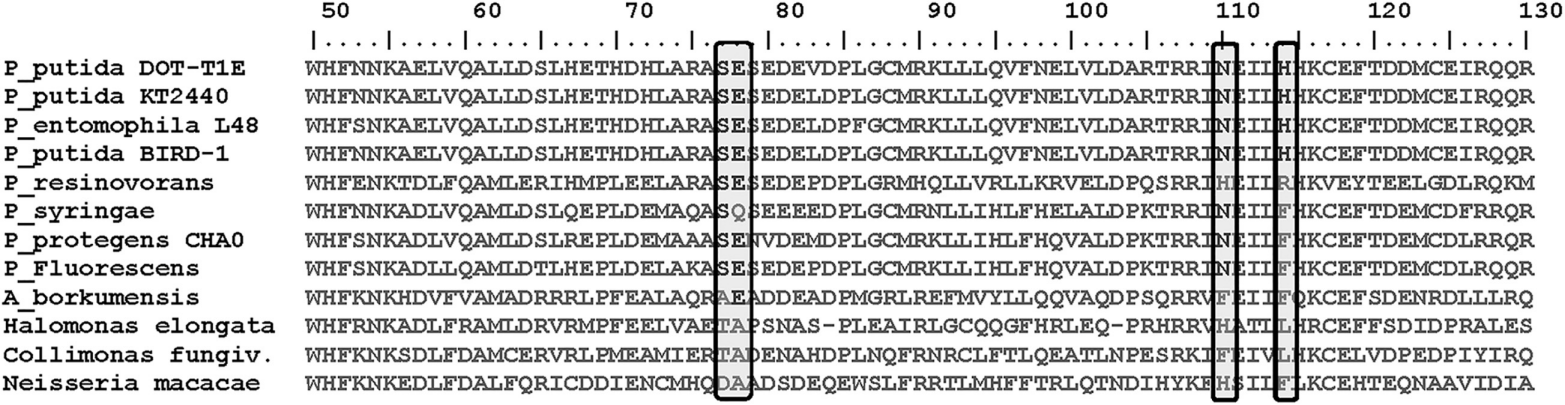
Sequence alignment of close homologues of TtgR (*Pseudomonas putida* DOT-T1E). Mutated residues (S77, E78, N110 and H114) are boxed and shaded.

Here, we have taken another step toward understanding the mechanism of interaction of TtgR with different antimicrobials and in elucidating the TtgABC efflux pump control mechanism. We have designed and produced several mutants in the TtgR regulator that are based on modeled protein structures, and tested the effect of ligand binding on protein stability, effector-regulator binding and effector mediated DNA-operator dissociation. Our results show that replacement of the target residues by alanine did not introduce appreciable conformational changes (Fig 2A, Table 1) but they did alter the stability of the TtgR dimer, affecting the interaction with the effectors being assayed. The stability of all of the TtgR variants increased upon effector binding. In general, the highest stability was detected in the presence of phloretin followed closely by, naringenin and chloramphenicol. Residues, S77 and E78 hold a strategic position in the effector portal formed by helix-α4 and helix-α7 [12], playing an important role not only in the ligand binding process, predominantly controlled by hydrophobic interactions, but in the stability of the protein as well. Residue S77, located in the C-term half of helix-α4, is highly conserved in the *Pseudomonas* genus while it is not conserved in other genus as confirmed by sequence alignment with the close homologues of TtgR (Fig 7). As shown by the DSC experiments (Fig 4), the thermal protein unfolding of the S77A mutant appeared to be much less cooperative than the other variants and the unfolding enthalpy decreased significantly. Consequently, this mutation destabilized the protein and could favor its aggregation, demonstrating the important role of the S77 residue in protein structural conservation. On the contrary, the E78A mutation, also located at helix-α4 and on the top of the pocket of the low affinity site, is less conserved than the S77 residue in the multi-alignment of the TtgR of species of the *Pseudomonas* genus (Fig 7). The stability of the E78A is the most affected upon effector binding due to direct interactions with effectors (Tables 2 and 3). This suggests a putative ‘assistant’ role for the E78 residue in the correct positioning of the ligand. This residue does not interact with naringenin, revealing an additional role to conserve the adequate conformation of helix-α4. TtgR^N110A^ is the most stable mutant and TtgR^H114A^ the least stable in the presence of any effector. Although, they are located in the same binding area, the H114 residue does not interact directly with effectors, and probably helps to maintain the conformation of the binding pocket or assists for the correct positioning of the effector. This fact matches well with the K_D_ values, obtained by binding for each effector; they are similar to those for the wild type (Table 4). Furthermore N110, a polar and uncharged amino acid, interacts directly with the polar regions of the effectors and appeared to play a role in effector binding. Both residues, H114 and N110 seem to play a possible instrumental role as effector sensors. Although, all of the mutants generated in this study were in residues outside of the HTH DNA binding domain, these mutations affected the DNA binding ability of the regulator suggesting an intramolecular chain of interactions that confer the regulatory properties of TtgR. In fact, for effectors that bind with high affinity, i.e., phloretin, there exists a correlation between the effector concentration and the level of induction of the operon *in vivo*, and the dissociation of TtgR from its target operator *in vitro* [11]. Effector concentration is not the only element critical for derepression in the case of low affinity effectors binding. This has also been described for others repressors such as FadR [27]. For all of the mutants analyzed in this study the K_D_ values for phloretin are smaller than those obtained for naringenin; therefore, it is expected that the mutant proteins would be released from target DNA by phloretin. However, our EMSA results showed total complex dissociation for TtgR^E78A^ and TtgR^H114A^ but only partial dissociation for TtgR^N110A^. In the presence of naringenin the opposite occurred; TtgR^E78A^ and TtgR^H114A^ gave partial dissociation from DNA while naringenin induced almost total complex dissociation for TtgR^N110A^. One interpretation of these results is that the binding of the effectors to TtgR leads to a global destabilization of the repressor which in turn leads to its release from the target operator.

## Conclusions

This study has deepened in the understanding of the interaction mechanism of TtgR to different antimicrobials. We have identified four residues (S77, E78, N110 and H114) in the protein binding pocket, whose mutations affect protein stability, the effector binding affinity and concomitantly the control of transcription of the TtgABC efflux pump. The mutation of residues H114 and S77 reduced notably the stability of the protein, while mutation E78A affected protein stability upon effector binding. Mutant N110A exhibited an increased stability, what was particularly evident in the presence of effectors. Additionally, all the mutants were affected in their dissociation from its target operator, in spite that the mutations laid in the effector binding pocket rather than in the DNA binding site. We suggest that derepression of TtgR from its target operator is mediated by global destabilization of the repressor, giving new relevant insights to deepen on the understanding of antimicrobial resistance.

## Supporting Information

S1 Fig Protein unfolding of TtgR WT and mutants in the presence of ligands monitored by CD ellipticity at 222 nm. (PDF)
