# Supplementary material for: Molecular Binding Mechanism of TtgR Repressor to Antibiotics and Antimicrobials
Source: PLoS One. 2015 Sep 30;10(9):e0138469. doi: 10.1371/journal.pone.0138469 (PMC4589371; doi:10.1371/journal.pone.0138469)
Supplement: S1 Fig — (PDF) [file pone.0138469.s001.pdf]

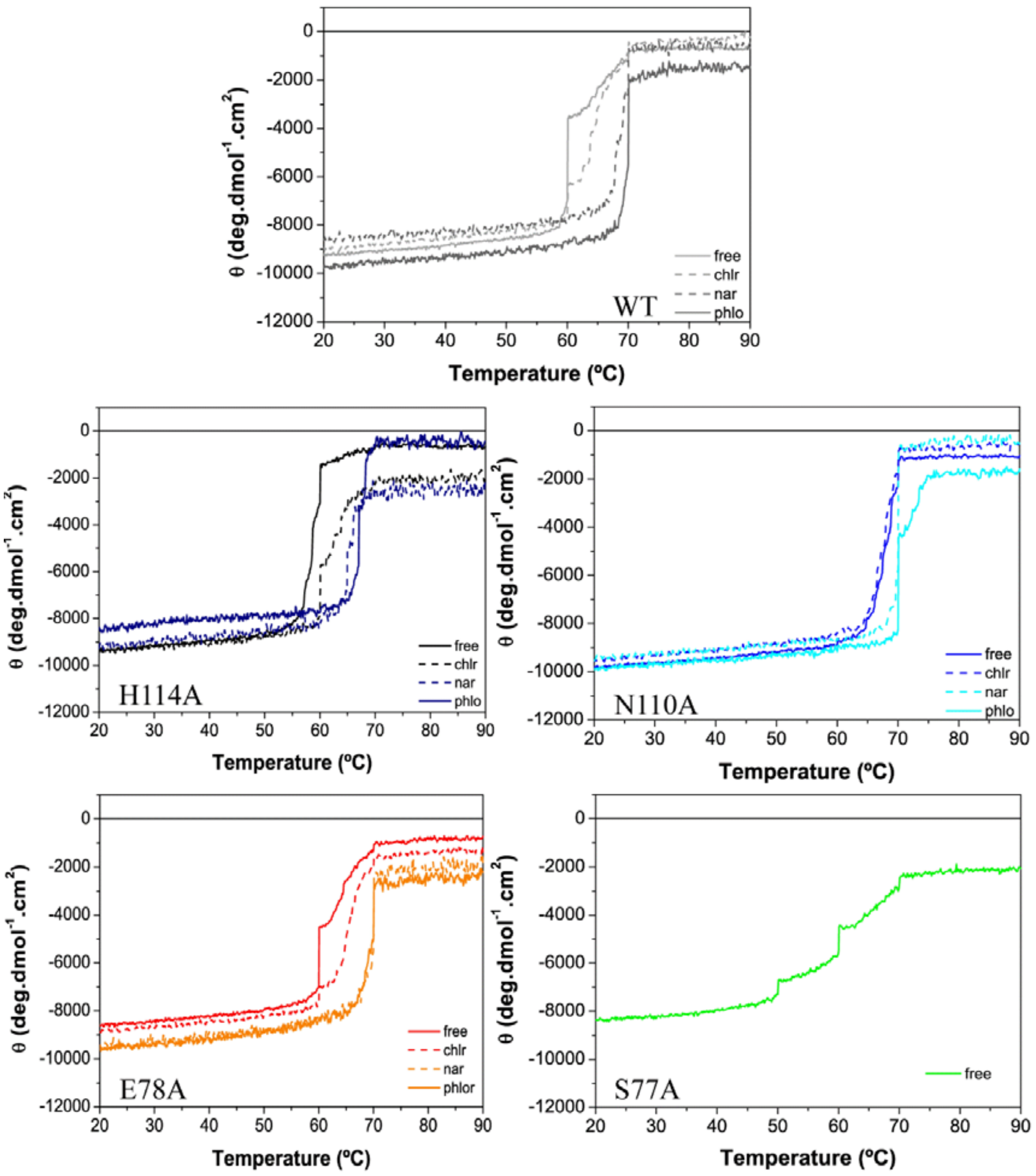

SI_Figure: Protein unfolding of TtgR WT and mutants in the presence of ligands monitored by CD ellipticity at 222 nm
